# RAB22A as a predictor of exosome secretion in the progression and relapse of multiple myeloma

**DOI:** 10.18632/aging.205565

**Published:** 2024-03-01

**Authors:** Bingjie Fan, Li Wang, Jishi Wang

**Affiliations:** 1 Department of Hematology, Affiliated Hospital of Guizhou Medical University, Guizhou Province Institute of Hematology, Guizhou Province Laboratory of Hematopoietic Stem Cell Transplantation Centre, Guiyang, China; 2 Clinical Medicine College of Guizhou Medical University, Guiyang, China

**Keywords:** RAB22A, exosome secretion, EMT, multiple myeloma, immune regulation

## Abstract

Background: Multiple myeloma (MM) is an incurable malignant plasma cell disease. We explored the role of RAB22A in exosome secretion, epithelial-mesenchymal transition (EMT) and immune regulation.

Methods: We obtained MM samples from Gene Expression Omnibus (GEO) data sets. We downloaded the “IOBR” package, and used the “PCA” and “ssGSEA” algorithms to calculate the EMT scores and exosome scores. The “CIBERSORT” package was used to analyze the infiltration of immune cells. We extracted the exosomes of mesenchymal stem cell (MSC) to verify the biological function of RAB22A.

Results: The expression level of RAB22A in smoldering multiple myeloma (SMM) and MM patients was significantly higher than that in normal people and monoclonal gammopathy of undetermined significance (MGUS) patients, and the expression level of RAB22A in relapse MM patients was significantly higher than that in newly diagnosed patients. The EMT scores and exosome scores of high RAB22A group were significantly higher than those of low RAB22A group, and the exosome scores of MSC in recurrent patients were significantly higher than those of newly diagnosed patients. In addition, the infiltration levels of monocyte, NK cells resting, eosinophils, T cells regulatory and T cells CD4 memory activated were positively correlated with RAB22A. After down-regulating the expression of RAB22A in MM-MSC, the secretion of exosomes decreased. Compared with the exosomes of MSC in si-RAB22A group, the exosomes in control group significantly promoted the proliferation of MM.

Conclusions: RAB22A is a potential therapeutic target to improve the prognosis of MM, which is closely related to exosome secretion, EMT and immune cell infiltration.

## INTRODUCTION

Multiple myeloma (MM) is an incurable malignant plasma cell disease of blood system, which is characterized by malignant expansion of monoclonal plasma cells in bone marrow or extramedullary position [1]. In recent years, the incidence of MM has increased, and the onset age is mostly over 65 years old. MM develops from an asymptomatic precancerous stage, first with MGUS, then with SMM, and finally with active multiple myeloma [2, 3]. Abnormal plasma cells of MM patients secrete monoclonal immunoglobulin, which leads to end organ damage and characteristic clinical manifestations such as hypercalcemia, renal failure, anemia and osteolytic bone destruction [4].

With the in-depth exploration of the pathogenesis and drug development of MM, many therapies, such as proteasome inhibitors, immunomodulatory drugs, antibody-drug conjugates (ADC) and chimeric antigen receptor (CAR) T-cell therapy, have been applied to MM, and achieved certain curative effects [5]. These drugs alone or in combination can improve the survival rate of newly diagnosed or relapsed MM patients from 25% in the mid-1970s to 55% [6]. The recent follow-up results of a phase III clinical trial for relapsed/refractory (RR) MM patients showed that patients treated with caffezomib, dyramuzumab and dexamethasone (KdD) had a higher realization rate of minimal residual disease-negative (MRD-) than patients treated with caffezomib and dexamethasone (Kd) (28% vs 9%), and the median PFS and OS of KdD group were significantly higher than those of Kd group [7]. Another real-world study included 200 RR MM patients who were treated with elotuzumab, pomalidomide and dexamethasone (EloPD). The overall response rate was 55.4%. Although the median PFS and OS were lower than those in the clinical trial of ELOQUENT-3, it still improved the survival rate of patients with RR MM [8]. The prognosis of MM has been greatly improved, but drug resistance and recurrence have seriously affected the economy and prognosis of patients. Some preclinical research to develop new drugs based on the target of MM may be a promising method. ISB 1342 is a CD38 × CD3 T-cell engager, which can effectively kill MM cell lines with low sensitivity to dartumumab *in vitro*, which may improve the survival time of patients with dartumumab resistance [9]. At present, MM can’t be cured, and patients still have many recurrences after remission, and the remission time after each recurrence is gradually shortened. With the development of cancer molecular biology and genomics, people’s understanding of tumor molecular phenotype is deepening. The strategy of tumor treatment has gradually changed into the era of precise targeted therapy for genomic changes. Therefore, finding new biomarkers is beneficial to insight into the pathogenesis and improve the prognosis of MM.

RAB22A is a member of RAS oncogene family, and its expression is increased in many cancers such as breast cancer [10], colorectal cancer [11], osteosarcoma [12] and liver cancer [13], and it is involved in the proliferation, migration and immune regulation of malignant tumors. As a small GTPase, RAB22A belongs to RAB5 subfamily, mainly located in early endosomes, Golgi bodies and late endosomes [14]. RAB proteins are involved in the regulation of vesicle transport, early endosome and exosome formation and endosome recovery. In one study, RAB22A mediated the migration and invasion of breast cancer cells by promoting exosome secretion, which can be regulated by targeting RAB22A through tumor suppressor gene miR-19b [15]. Interestingly, another study found that RAB22A-mediated autophagosome containing STING fused with early endosomes, resulting in a new organelle, Rafeesome, which promoted the secretion of vesicles packaging STING into tumor microenvironment, thus mediating anti-tumor immunity [16]. RAB22A can mediate EMT, promote tumor cell migration and lead to tumor progression. MCF2L-AS1 promoted the EMT of colorectal cancer by targeting the miR-105-5p/RAB22A axis, thus making the malignant progression of colorectal cancer [17]. DNMT1 regulated the DNA methylation in the promoter region of miR-211, decreased the expression level of miR-211 in melanoma cells and increased the expression level of its downstream target gene RAB22A, thus promoting the EMT process [18]. In addition, RAB22A, as an immunomodulatory factor, plays an immune role in many tumors. RAB22A controlled MHC-I intrinsic distribution, recycling and trafficking to dendritic cell (DC) phage, which played an extremely important role in the antigen cross-presentation reaction of DC [19]. In another study, RAB22A was significantly up-regulated in hepatocellular carcinoma, which was significantly related to the infiltration of various immune cells and the gene expression of immune checkpoints, and may lead to poor prognosis of hepatocellular carcinoma by regulating immune function [13]. However, RAB22A is rarely studied in hematological tumors, and there is no related research in MM at present. Therefore, it urges us to explore the effects of RAB22A in MM on extracellular vesicle secretion, EMT and immune regulation.

In this study, we analyzed the difference of the expression level of RAB22A between normal people and MM patients, between newly diagnosed and relapsed MM patients by including MM samples in GEO databases. We also explored the related biological functions of high and low RAB22A expression groups through EMT gene set and exosome gene set. In addition, we analyzed the correlation between RAB22A and immune cell infiltration and immune checkpoint genes. Finally, we made a preliminary verification in the clinical samples of our center. Our study explored the effect of RAB22A expression in MM on EMT, exosome secretion and immune regulation, which has guiding significance for the prognosis prediction and drug target development of MM.

## MATERIALS AND METHODS

### Source of data

We obtained 78 samples from GSE5900 dataset in GEO database, including 22 normal people, 44 MGUS patients and 12 SMM patients. We also extracted the data of 414 newly diagnosed MM patients from GSE4581 dataset for the analysis of RAB22A expression level and biological function. In addition, we extracted the MSCs expression data of 12 newly diagnosed and 9 relapsed MM patients from GSE146649 dataset, and analyzed the expression levels of RAB22A and exosome-related genes. We use “sva” package to correct batch effect in these data sets.

### Analysis of differentially expressed genes (DEGs) of RAB22A

We extracted the RAB22A expression level of 414 newly diagnosed MM patients with GSE4581 by using the “limma” package, and divided the samples into high RAB22A expression group and low RAB22A expression group according to the median value. The heatmap and volcano plot showed the DEGs in the high and low RAB22A expression groups. Gene Ontology (GO) enrichment analysis and Kyoto encyclopedia of genes and genomes (KEGG) enrichment analysis were carried out to further explore the potential biological functions.

### Correlation analysis of RAB22A with EMT, exosome, immune checkpoints and m6A related genes

In order to further clarify the biological function of RAB22A in MM, we selected EMT, exosome, immune checkpoint and m6A related gene sets. We obtained EMT-related genes from the website of dbEMT2.0, and intersected EMT genes with the DEGs related to RAB22A. Exosome gene set is divided into three modules, including genes related to extracellular vesicle, exosome marker genes and genes regulating exosome secretion [20–23] (see in Supplementary Table 1). We selected 25 immune checkpoint genes (Supplementary Table 2). In addition, we selected 14 m6A-related genes for analysis (GSE4581 dataset lacks some m6A-related genes), as shown in Supplementary Table 3. Wilcox. test was used to calculate the correlation between gene sets and RAB22A, and the results were presented by boxplot and heatmap.

### Analysis of immune cell infiltration

We used the “CIBERSORT” package for immune cell infiltration analysis. The degree of immune cell infiltration in high and low RAB22A expression groups was analyzed, and the “ggplot2” and “ggpubr” packages were used to draw borplot and barplot. In addition, we further drew scatterplots between the expression level of RAB22A and the infiltration level of immune cells.

### Calculation of EMT and exosome scores

We downloaded the “IOBR” package with “calculate_sig_score” function and used “PCA” and “ssGSEA” algorithm to calculate EMT scores and exosomes scores of different RAB22A groups.

### Clinical samples and cell lines

We collected the clinical information and bone marrow samples of 71 normal controls and 30 newly diagnosed MM patients in the hematology department of the Affiliated Hospital of Guizhou Medical University. All participants obtained informed consent, and the research was approved by the ethics committee of the Affiliated Hospital of Guizhou Medical University. Human MM cell lines RPMI 8226 and U266 were provided by Hematopoietic Stem Cell Transplantation Center Laboratory in Guizhou province. All human cell lines were tested for mycoplasma contamination and verified by short tandem repeats. Ficoll gradient centrifugation was used to separate MSCs, and the 2-4 generation cells were selected for further experiments. Cell lines were cultured in RPMI-1640 (Gibco, USA) medium with 10% fetal bovine serum (FBS), penicillin (100 units/mL) and streptomycin (100 mg/mL) at 37° C and 5% CO_2_ with saturating humidity. MSCs were cultured in complete medium of bone marrow mesenchymal stromal cells (BM-MSCs) (OriCell, China), and 48 hours before exosomes were extracted, they were changed to medium containing exosome-depleted FBS (OriCell, China).

### Quantitative real-time PCR (qRT-PCR)

Total RNA was extracted from cells by using Trizol reagent (Invitrogen, USA) according to the manufacturer’s instruction. SYBR Green PCR Master Mix (MCE, China) and PRISM 7500 real-time PCR detection system (ABI, USA) were used for real-time PCR detection. The primers used in this study are as follows: for RAB22A, forward: 5′-TACCAAAGAGGCAAAGCATGT GCG-3′, reverse: 5′-AGACACCATGCAATCACCAACAGC-3′; for β-actin, forward: 5′-GAGACCTTCAACACCCCAGC-3′, reverse:5′-ATGTCACGCACGATTTCCC-3′. The relative expression of mRNA was normalized to β-actin and calculated by 2^-ΔCT^.

### Western blotting analysis

Western blotting was used to detect the expression of RAB22A in normal people and MM patients, and normalized it to β-actin. BCA protein assay kit (Pierce, Hercules, USA) was used to determine the protein concentration. The protein samples were loaded on 10% SDS-PAGE gel and transferred to PVDF membranes. After being sealed with 5% skim milk, the membrane was incubated overnight at 4° C with the following antibodies: anti-RAB22A (1:500, Affinity, China) and anti β-actin (1:3000, Proteintech Group, China). Protein bands were observed using ECL kit (7 Sea Biotechnology, China).

### Lentiviral transduction

Human RAB22A-RNAi was purchased from Genechem Co., Ltd. (Shanghai, China). Transfection of RAB22A was performed according to the manufacturer’s protocol. Cells (RPMI 8226, U266 cell line and MSCs) transfected with empty vector (EV) were used as controls. After amplification and maintenance in culture medium, stable RPMI 8226, U266 cell lines and MSCs expressing si-RAB22A were selected by puromycin (1ug/ml).

### Extraction and identification of exosomes

According to the manufacturer’s instructions, the supernatant of MSCs cultured in medium containing exosome-depleted FBS was collected. Exosome concentration solution (MCE, China) was added to MSC supernatant, and crude exosomes were obtained after centrifugation. Purified exosomes were obtained by centrifugation with exosome purification filter (MCE, China). Western blotting was used to identify exosome surface markers CD63 (Immunoway, USA) and TSG101 (Immunoway, USA).

### Immunofluorescence

The exosomes resuspended in 200ul phosphate buffer saline were incubated with PKH26 (Solarbio, China) in the dark for 5 minutes. MM cell lines were inoculated into 24-well plates, and the labeled MSCs-derived exosomes were added. After 24 hours, the cells were collected and fixed with formaldehyde, then 4’,6-diamidino-2-phenylindole (DAPI) was added for nuclear staining. After that, the cells were observed under a fluorescence microscope.

### Cell activity detection

Cells were inoculated into a 96-well plate, and exosomes filtered with a 0.22um filter were added. Cell Counting Kit-8 (CCK-8) was added at different time points and incubated at 37° C for 2 hours. The absorbance at 450nm was measured by spectrophotometer.

### Statistical analysis

R software package (version 4.1.3) was used for statistical analysis and visualization of results. Shapiro-Wilk normality test was used to test the normality of variables. Unpaired student’s t-test was used to evaluate the statistical significance of the data that conforms to the normal distribution. Using 1: 1 properness score matching (PSM) to eliminate the bias between the control group and the newly diagnosed MM group. P<0.05 indicated statistical significance.

### Data availability statement

All data supporting the results of this study can be obtained from the corresponding authors according to reasonable requirements. The datasets presented in this study can be found in online repositories. The names of the repository can be found below: https://portal.gdc.cancer.gov.

## RESULTS

### The expression of RAB22A increased in MM

**Figure 1:**
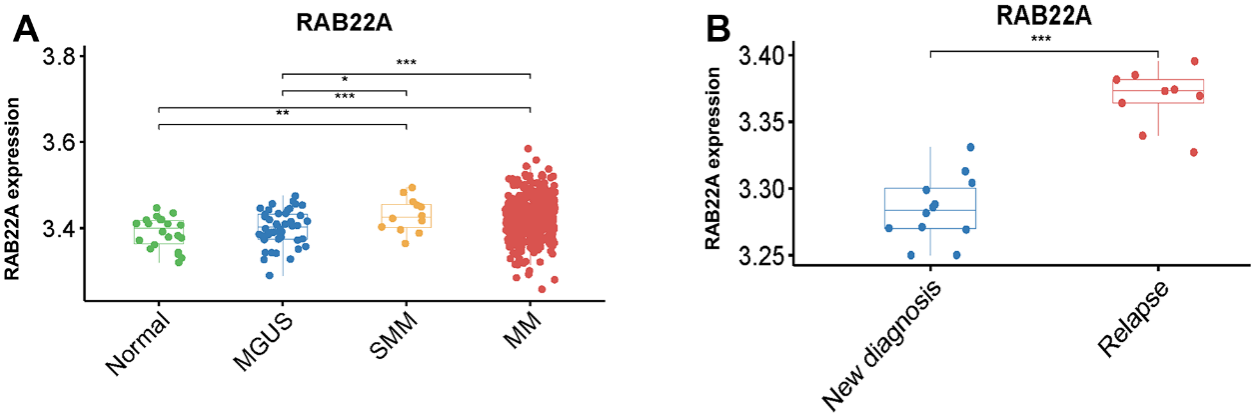
**Differential analysis of expression level of RAB22A.** (**A**) Differential expression analysis of RAB22A in normal people, MGUS, SMM and MM patients. (**B**) Differential expression of RAB22A in MSC of newly diagnosed and relapsed MM patients.

Firstly, we explored the expression level of RAB22A in normal people, MGUS, SMM and MM patients, and found that the expression level of RAB22A in SMM and MM patients was significantly higher than that in normal people and MGUS patients (Figure 1A). In order to further clarify the prognostic role of RAB22A in MM, we analyzed the expression level of RAB22A in newly diagnosed and relapsed MM patients. The results showed that the expression level of RAB22A in relapsed MM patients was significantly higher than that in newly diagnosed MM patients (Figure 1B). These results showed the prognostic value of RAB22A for MM patients, which urged us to further explore the biological function of RAB22A.

### Analysis of DEGs related to RAB22A

**Figure 2:**
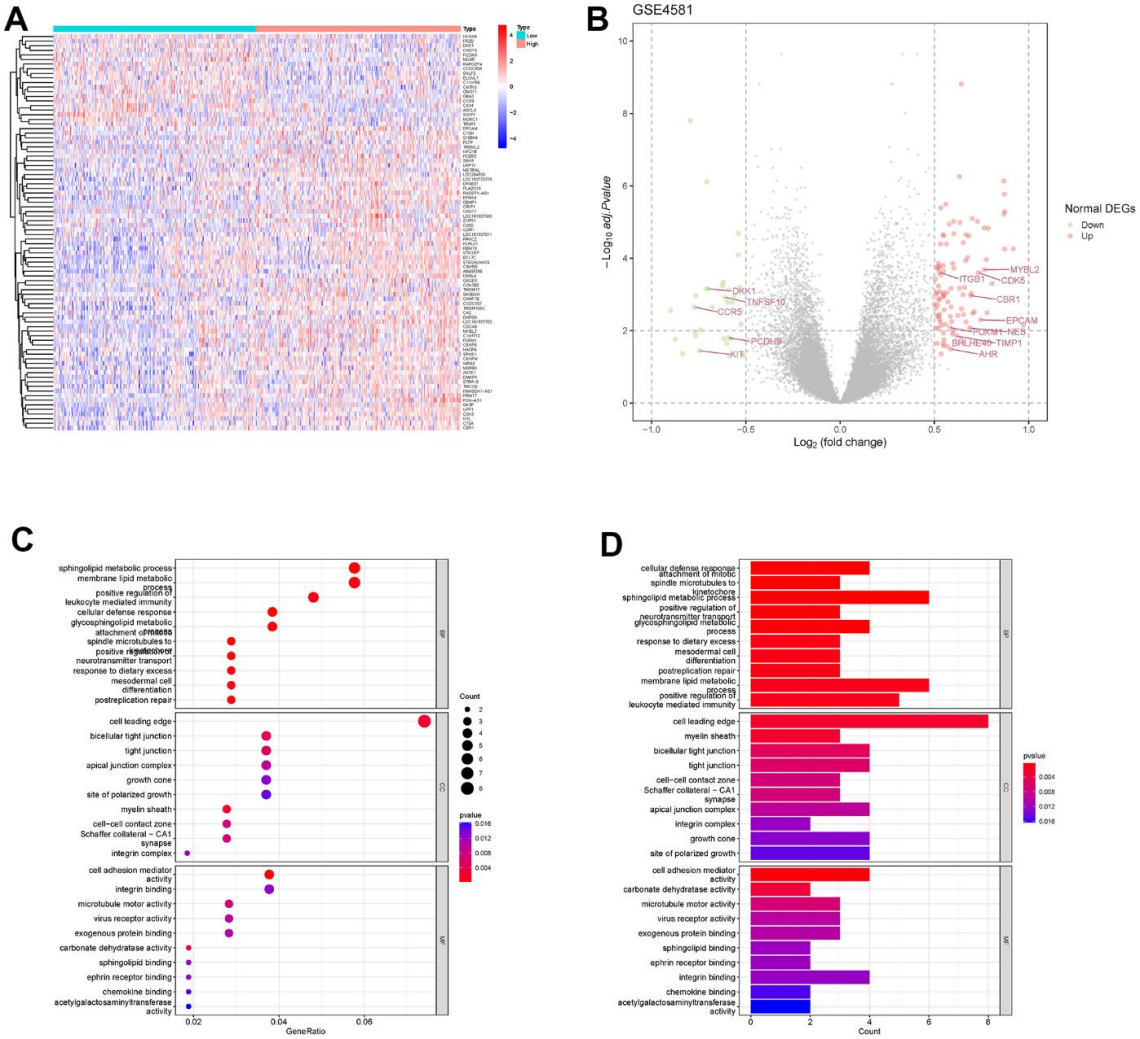
**Differentially expressed genes and enrichment analysis in different RAB22A groups.** (**A**, **B**) The differentially expressed genes of high and low RAB22A groups were presented by heatmap and volcano plot. (**C**, **D**) GO enrichment analysis was performed on differentially expressed genes.

According to the median value of RAB22A in GSE4581 dataset, we divided the samples into high RAB22A expression group and low RAB22A expression group. The DEGs of the two groups were presented in the heatmap and volcano plot, and we marked EMT genes (Figure 2A, 2B) in the volcano plot. Then, we carried out GO and KEGG enrichment analysis on the DEGs. The results of GO enrichment analysis showed that biological process (BP) was mainly related to membrane lipid metabolic process, sphingolipid metabolic process and positive regulation of leukocyte mediated immunity. Cellular component (CC) was mainly related to cell leading edge, bicellular tight junction and apical junction complex. And Molecular function (MF) was related to cell adhesion mediator activity, integrin binding and microtubule motor activity (Figure 2C, 2D). KEGG results are shown in the Supplementary Figure 1. These results suggest that DEGs may be involved in intercellular communication, migration and immunity of MM.

### Correlation between RAB22A and EMT

**Figure 3:**
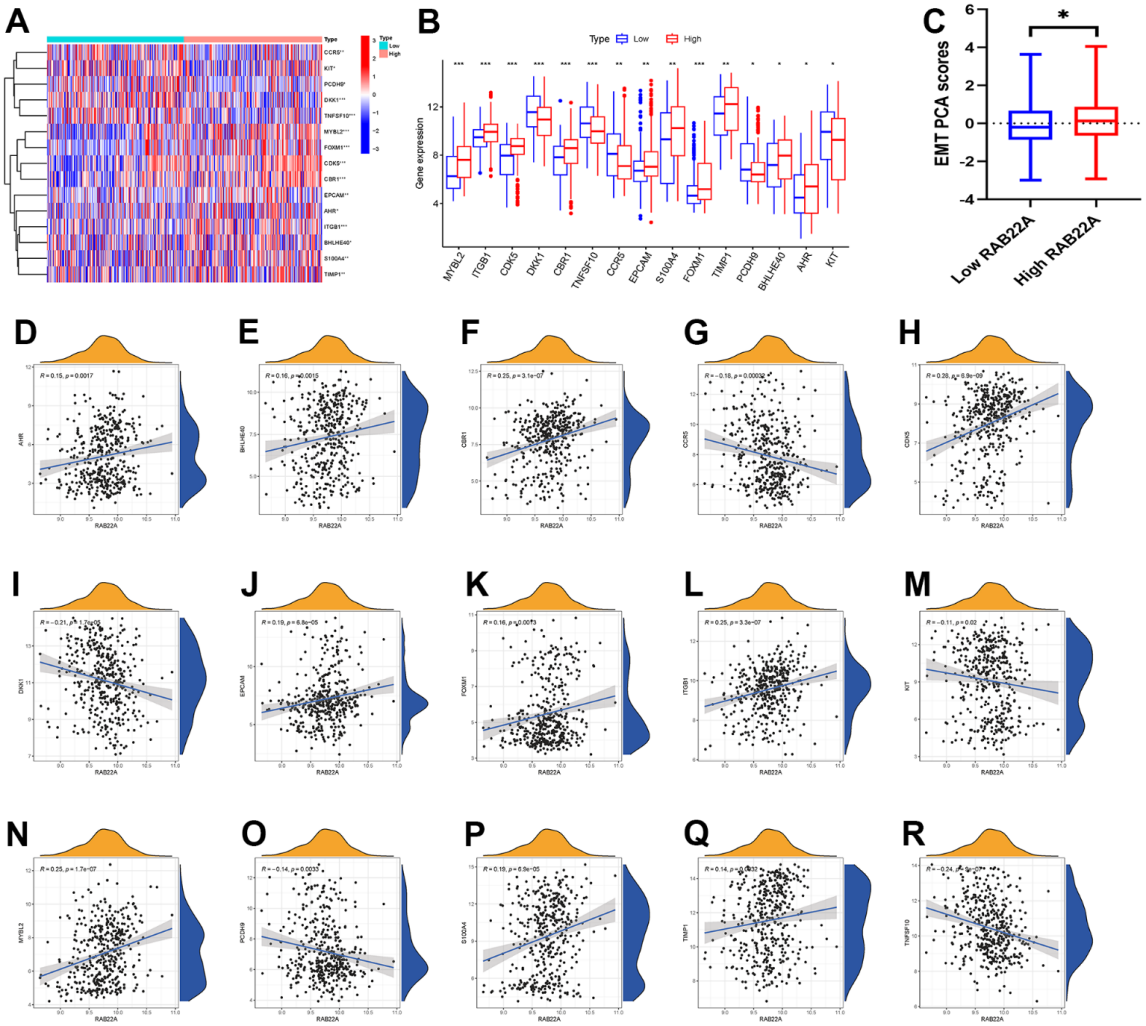
**Correlation analysis between RAB22A and EMT.** (**A**, **B**) EMT-related genes with significantly different expression levels between high and low RAB22A groups. (**C**) EMT scores of high and low RAB22A groups were calculated. (**D**–**R**) Scatterplots and correlation of RAB22A and EMT-related genes.

We obtained the DEGs in the high and low RAB22A expression groups, and intersected them with the EMT gene set to obtain 15 EMT genes related to RAB22A. We showed the EMT genes expression levels (Figure 3A, 3B) among different RAB22A expression groups by using heatmap and boxplot. The results showed that the expression levels of MYBL2, ITGB1, CDK5, CBR1, EPCAM, S100A4, FOXM1, TIMP1, BHLHE40 and AHR in high RAB22A expression group were higher than those in low RAB22A expression group, while the expression levels of DKK1, TNFSF10, CCR5, PCDH9 and KIT in high RAB22A expression group were lower. In addition, we drew scatterplots of the correlation between RAB22A and EMT genes (Figure 3D–3R). Finally, we calculated the EMT scores of different RAB22A groups through the “IOBR” package, and the results showed that the EMT scores of high RAB22A expression group were significantly higher than those of low RAB22A expression group (Figure 3C).

### Correlation between RAB22A expression and exosome scores

Exosomes contain microRNAs (miRNAs), mRNAs, proteins, lipids and so on, which are used for information transmission between cells. MSCs are an important part of bone marrow microenvironment, and BM-MSCs can mediate drug resistance of many hematological neoplasms [24]. BM-MSCs can influence tumor cells through various information interaction ways, and induce tumor cells to escape the killing of chemotherapy drugs. Previous studies have found that exosomes secreted by BM-MSCs in tumor microenvironment affect the progression of leukemia by transporting microRNA-7-5p into acute myeloid leukemia (AML) cells [25]. Therefore, the exosomes secretion of MSCs may be an important factor leading to the progress of hematological tumors, which urges us to pay attention to the genes regulating exosomes secretion.

**Figure 4:**
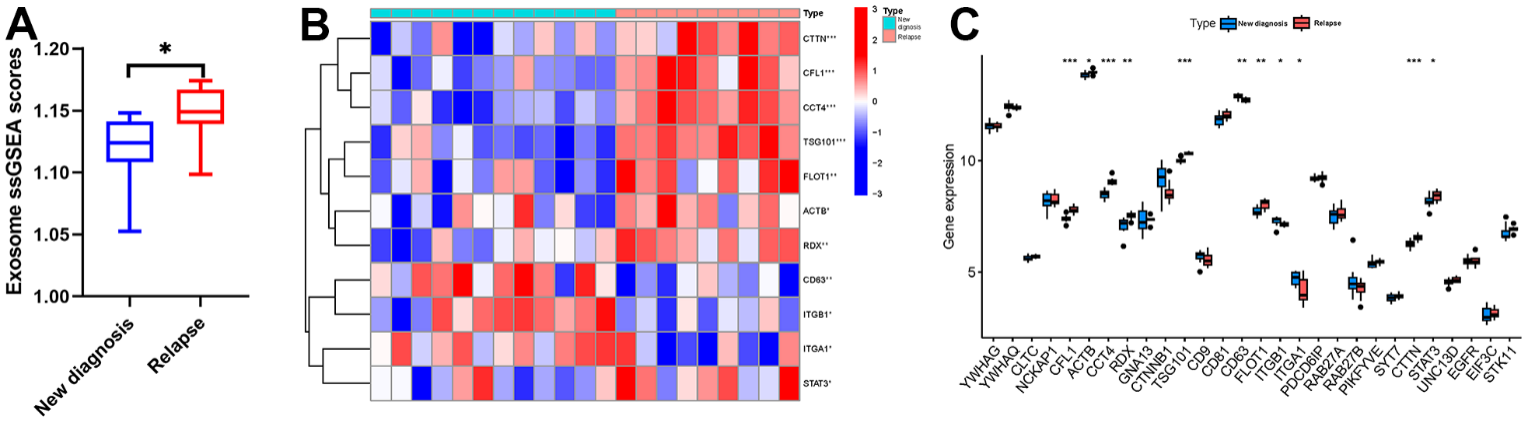
**Relationship between MM disease state and exosomes secretion.** (**A**) Exosome scores of MSCs in newly diagnosed and recurrent patients. (**B**, **C**) Heatmap and boxplot showed the expression level of exosome-related genes in MSCs of newly diagnosed and relapsed MM patients.

**Figure 5:**
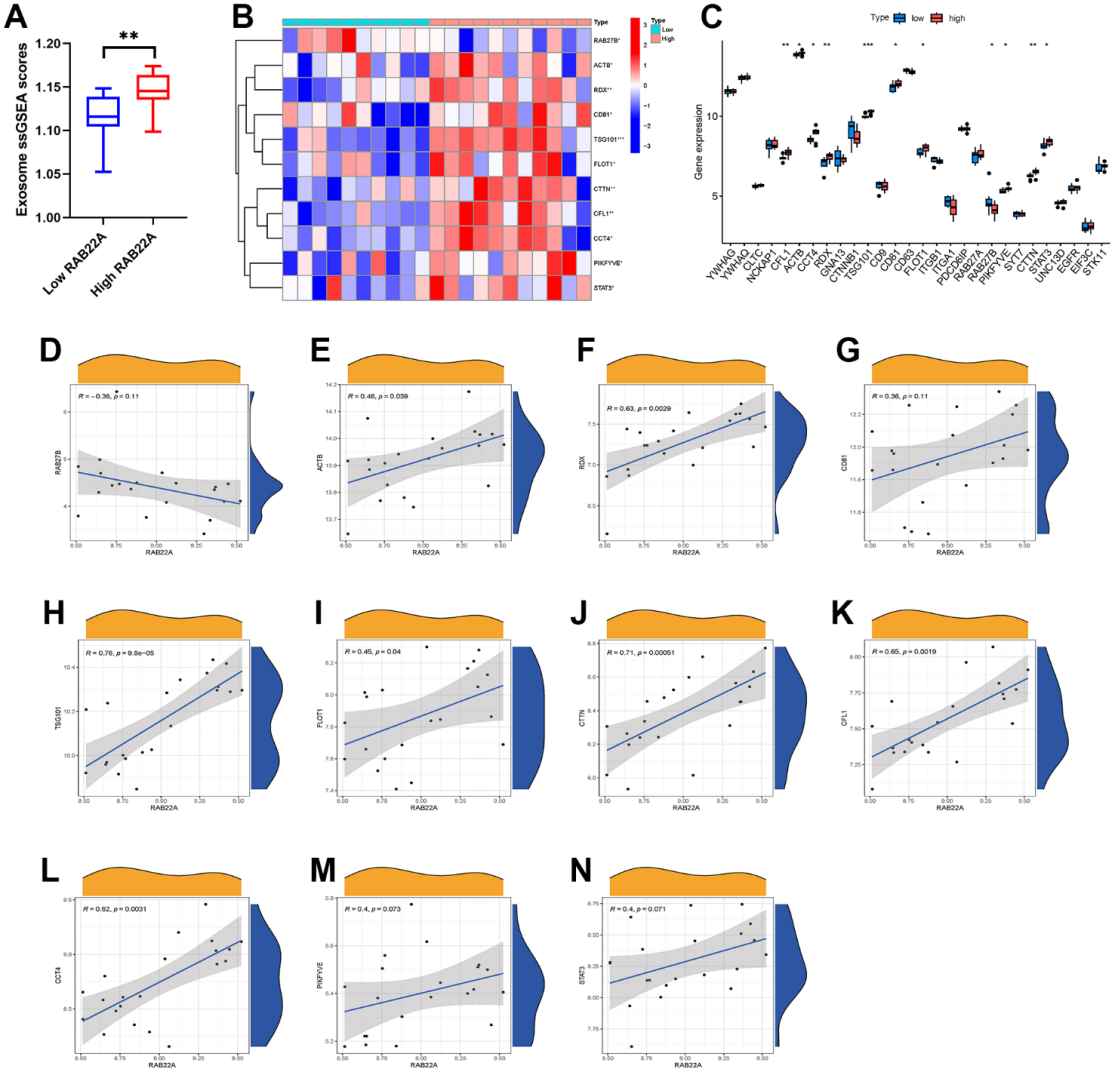
**Relationship between RAB22A and exosomes secretion.** (**A**) Exosome scores in high and low RAB22A groups. (**B**, **C**) Expression level of exosome-related genes in high and low RAB22A groups. (**D**–**N**) Correlation and scatterplots between RAB22A and exosome-related genes.

We extracted the expression data of MSCs from 12 newly diagnosed MM patients and 9 relapsed MM patients in GSE146649 dataset, and calculated the exosome scores with “ssGSEA” algorithm. The results showed that the exosome scores of MSC in relapsed patients was significantly higher than that in newly diagnosed patients (Figure 4A). Then, we further analyzed the expression levels of exosome-related genes in newly diagnosed and relapsed patients. The results showed that the expression levels of CFL1, ACTB, CCT4, RDX, TSG101, FLOT1, CTTN, and STAT3 in relapsed patients were significantly higher than those in newly diagnosed patients (Figure 4B, 4C). In order to further clarify the effect of RAB22A on exosomes secretion, we extracted the expression data of RAB22A from newly diagnosed and relapsed patients, and divided the samples into high and low RAB22A expression groups according to the median value. The results showed that the exosome scores of high RAB22A expression group was significantly higher than that of low RAB22A expression group (Figure 5A). Heatmap and boxplot showed that the expression levels of exosome-related genes in high RAB22A expression group and low RAB22A expression group. The results showed that the expression levels of CFL1, ACTB, CCT4, RDX, TSG101, CD81, FLOT1, PIKFYVE, CTTN and STAT3 in high RAB22A expression group were significantly higher than those in low RAB22A expression group (Figure 5B, 5C). Finally, we showed the correlation between the expression level of RAB22A and exosome genes by scatterplots (Figure 5D–5N).

### The correlation between RAB22A and immune cell infiltration and immune checkpoint genes

**Figure 6:**
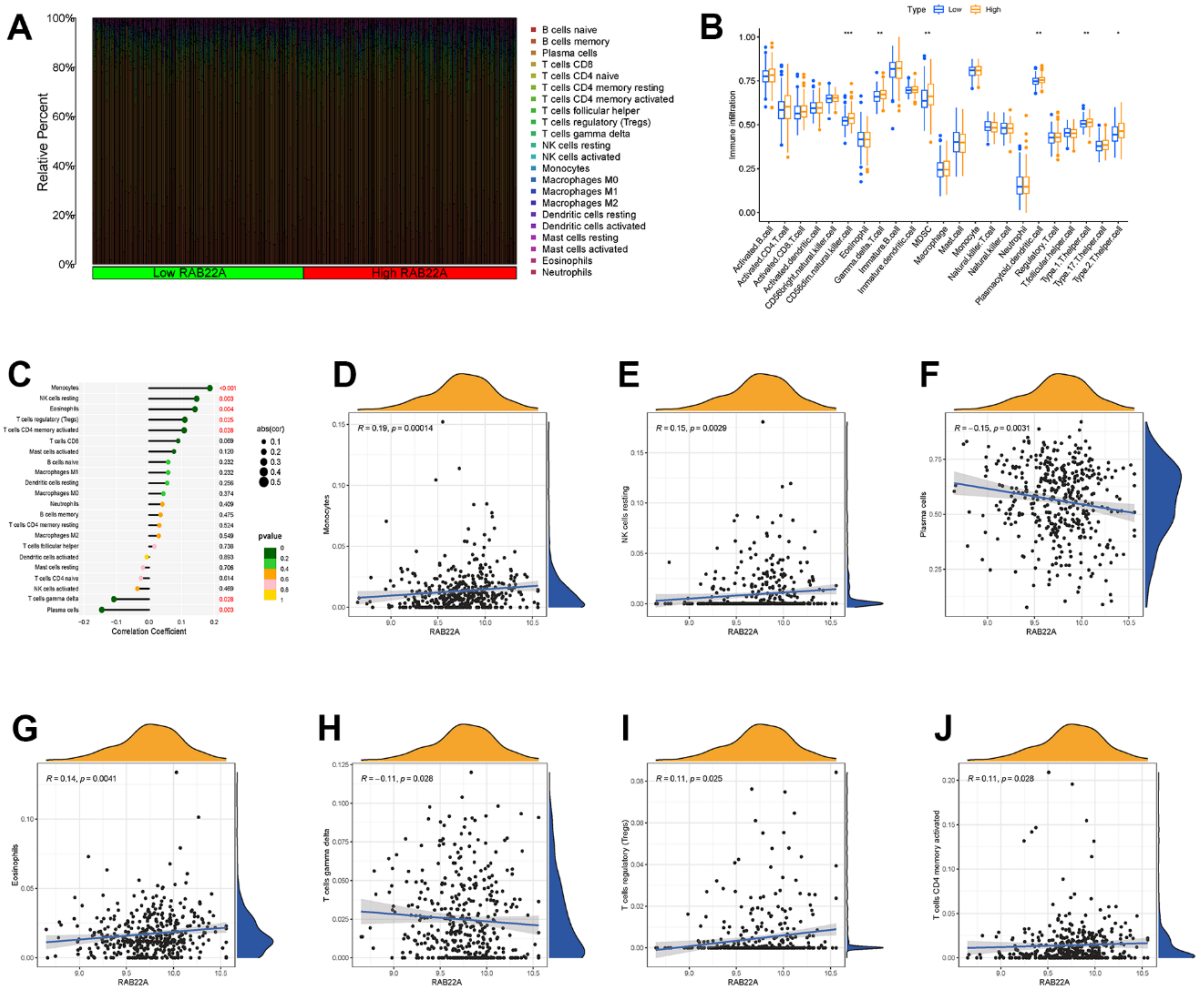
**Relationship between RAB22A and immune cell infiltration.** (**A**, **B**) Infiltration level of immune cells in high and low RAB22A groups. (**C**) Correlation between RAB22A and infiltration level of immune cells. (**D**–**J**) Scatterplots of RAB22A and immune cell infiltration.

We explored the infiltration levels of immune cells in different RAB22A expression groups in the samples of GSE4581 data set. The infiltration levels of CD56 dim natural killer cell, gamma delta T cell, myeloid-derived suppressor cell (MDSC), plasmacytoid dendritic cell, type 1 T helper cell and type 2 T helper cell in the high RAB22A expression group were significantly higher than those in the low RAB22A expression group (Figure 6A, 6B). In order to further clarify the correlation between RAB22A and immune cell infiltration, we drew scatterplots. The results showed that monocyte, NK cells resting, eosinophils, T cells regulatory (Treg) and T cells CD4 memory activated were positively correlated with RAB22A. While plasma cells and T cells gamma delta were negatively correlated with RAB22A (Figure 6C–6J).

**Figure 7:**
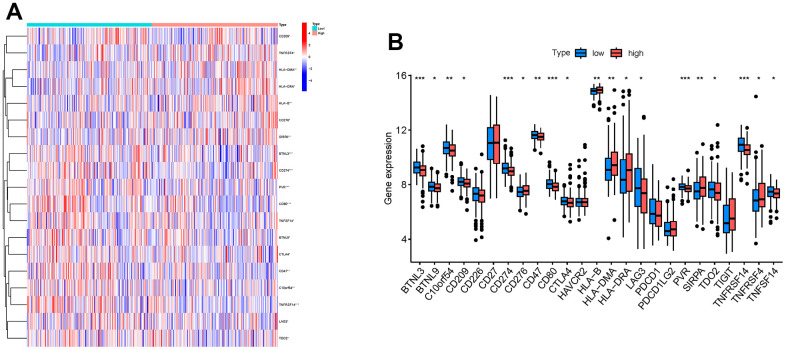
**Relationship between RAB22A and immune checkpoint genes.** (**A**) Heatmap showed the gene expression level of immune checkpoint genes in high and low RAB22A groups. (**B**) Boxplot showed the gene expression level of immune checkpoint genes in high and low RAB22A groups.

In addition, we selected 25 immune checkpoint genes and analyzed the expression levels of these genes in high and low RAB22A expression groups. The results showed that the expression levels of BTNL3, BTNL9, C10orf54, CD209, CD274, CD47, CD80, CTLA4, LAG3, PVR, TDO2, TNFRSF14 and TNFSF14 in the high RAB22A group were lower than those in the low RAB22A group, while CD276, HLA-B, HLA-DMA, HLA-DRA, SIRPA and TNFRSF4 were higher in the high RAB22A group (Figure 7A, 7B).

### RAB22A was associated with m6A RNA methylation regulators

**Figure 8:**
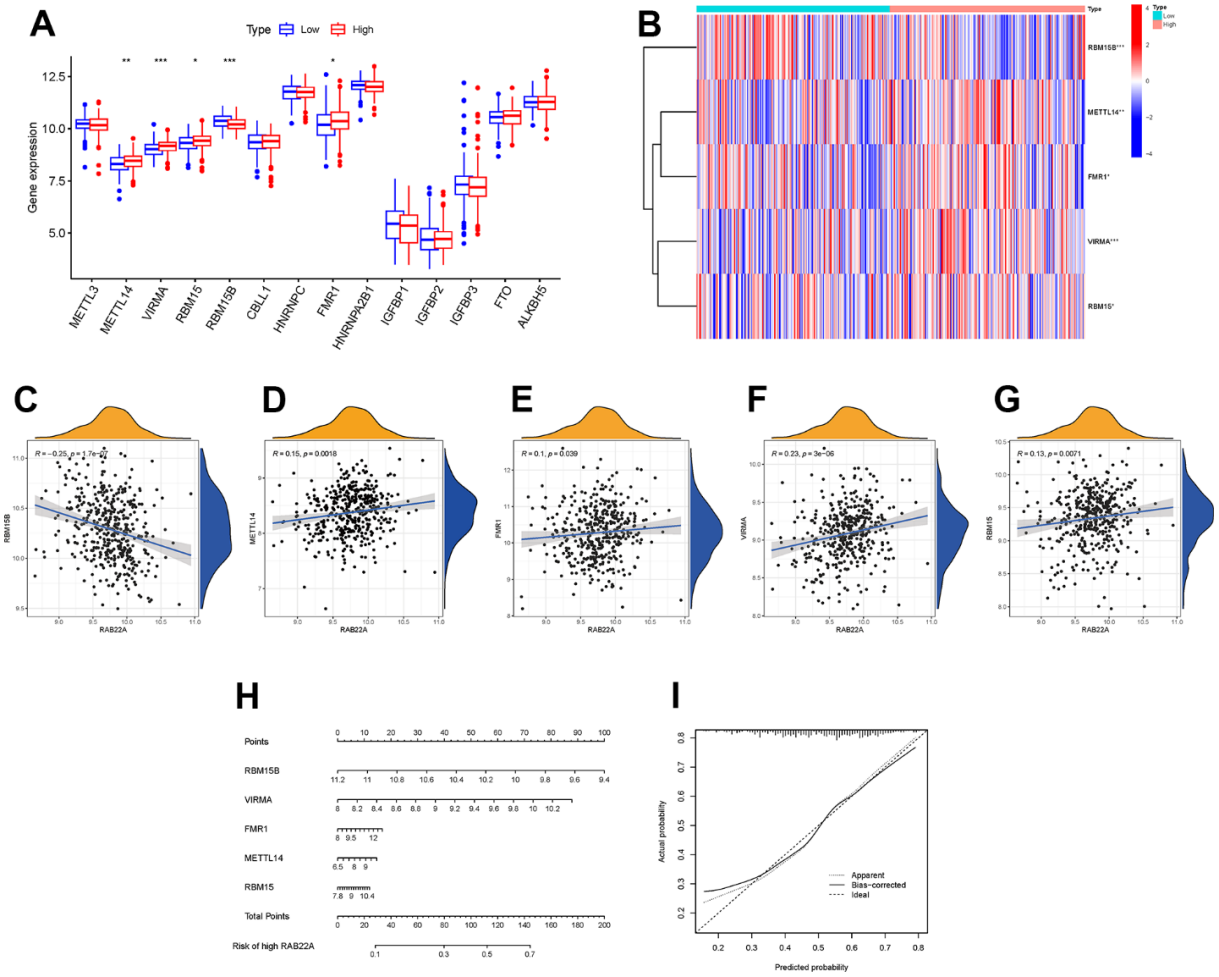
**Relationship between RAB22A and m6A methylation.** (**A**, **B**) Expression level of m6A methylation-related genes in different RAB22A groups. (**C**–**G**) Correlation and scatterplots between RAB22A and m6A methylation-related genes. (**H**, **I**) Nomogram and calibration curve of the risk of expressing RAB22A.

M6A RNA methylation is the most common internal modification in mammalian mRNA, which plays a key biological role by regulating important cellular processes, especially in cancer, where the modification disorder of m6A methylation is often observed. We analyzed the expression levels of m6A-related genes in different RAB22A expression groups. The results showed that the expression levels of METTL14, VIRMA, RBM15 and FMR1 in high RAB22A expression group were significantly higher than those in low RAB22A expression group (Figure 8A, 8B), and the scatterplots showed that these genes were positively correlated with RAB22A (Figure 8C–8G). In addition, we constructed a nomogram (Figure 8H) based on the m6A methylation genes significantly related to RAB22A, which was used to predict the risk value of overexpression of RAB22A. The calibration curve showed that the nomogram had good predictive ability (Figure 8I).

### Drug sensitivity testing

**Figure 9:**
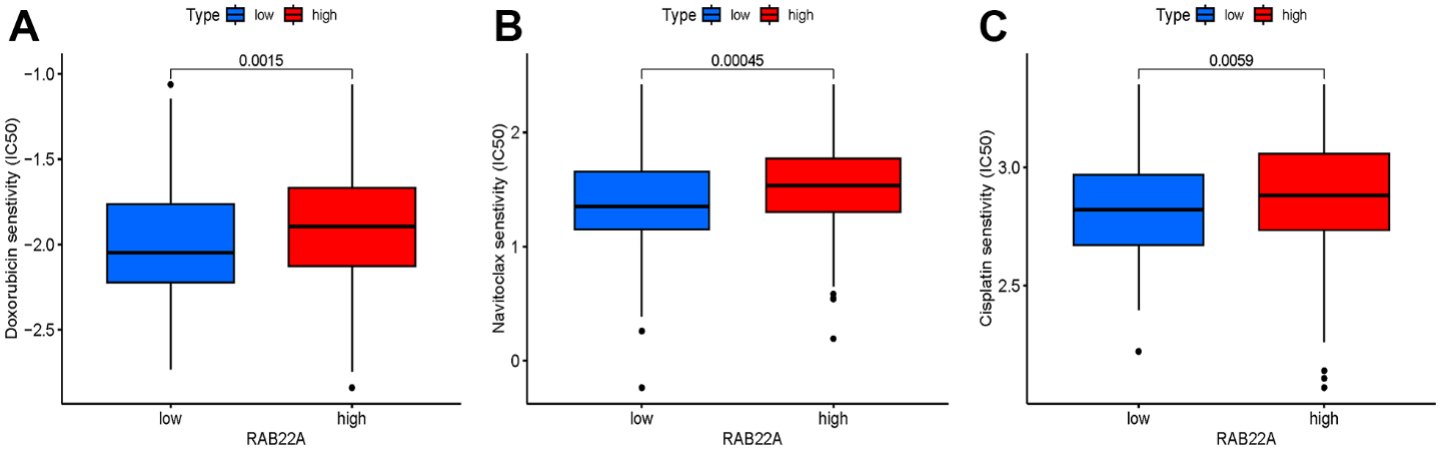
(**A**–**C**) Drugs with significantly different IC50 values between high and low RAB22A groups.

We screened sensitive drugs according to the difference of half maximum inhibition concentration (IC50) between high and low RAB22A expression groups. After consulting the literature, we focused on the drugs that have been used or have prospects in MM. It was worth noting that the IC50 values of doxorubicin, bcl-2 inhibitor (Navitoclax) and cisplatin in low RAB22A expression group were significantly lower than those in high RAB22A expression group (Figure 9A–9C).

### The effect of RAB22A on exosome secretion and cell proliferation

**Figure 10:**
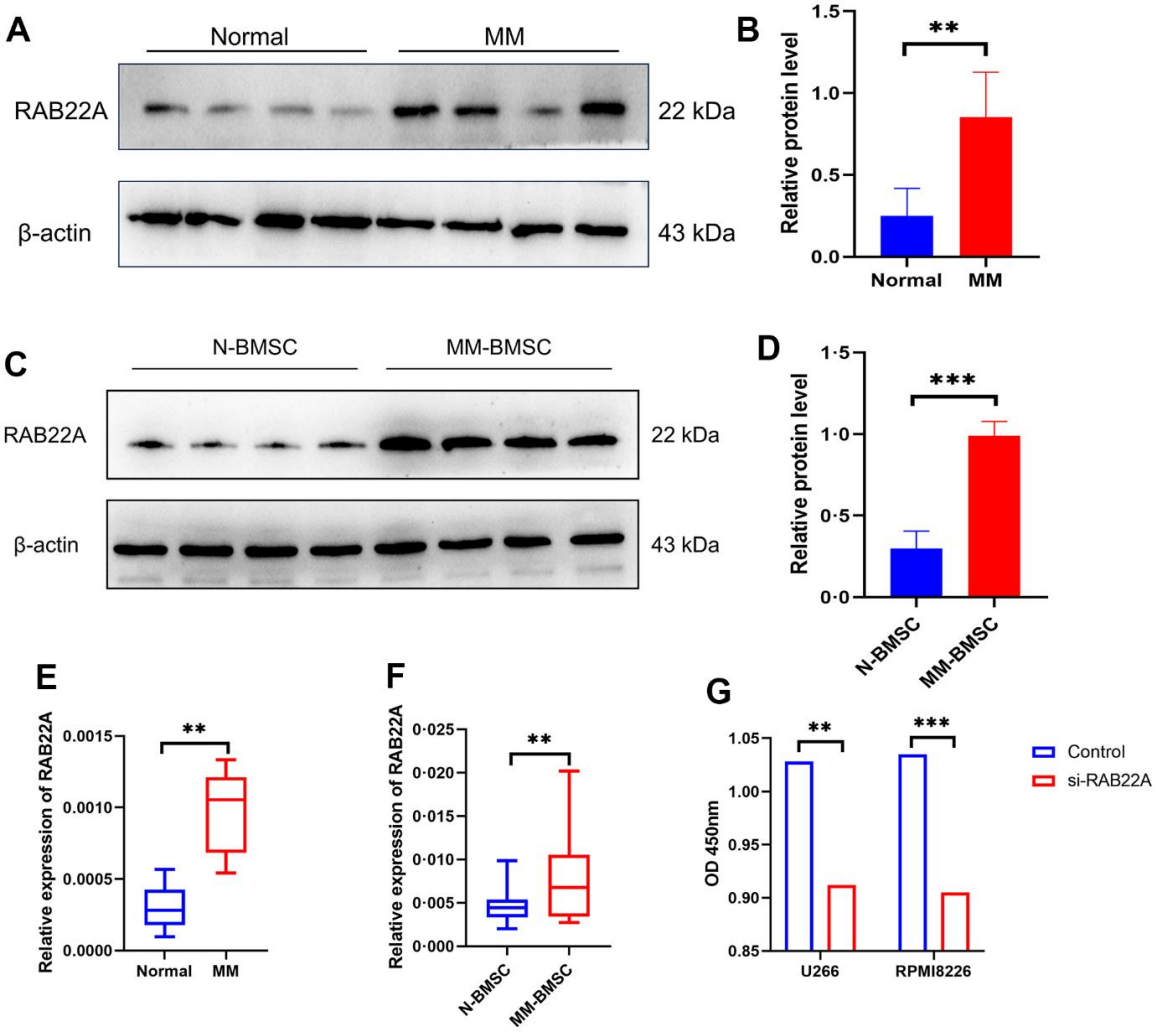
**To verify the expression level of RAB22A in clinical samples and cell lines.** (**A**–**D**) Western blotting results showed that the expression level of RAB22A protein in MM patients was significantly higher than that in normal people. (**E**, **F**) qRT-PCR results showed that the expression level of RAB22 gene in MM patients was significantly higher than that in normal people. (**G**) CCK-8 assay showed that the OD value decreased after down-regulating the expression of RAB22A in MM cell lines.

**Figure 11:**
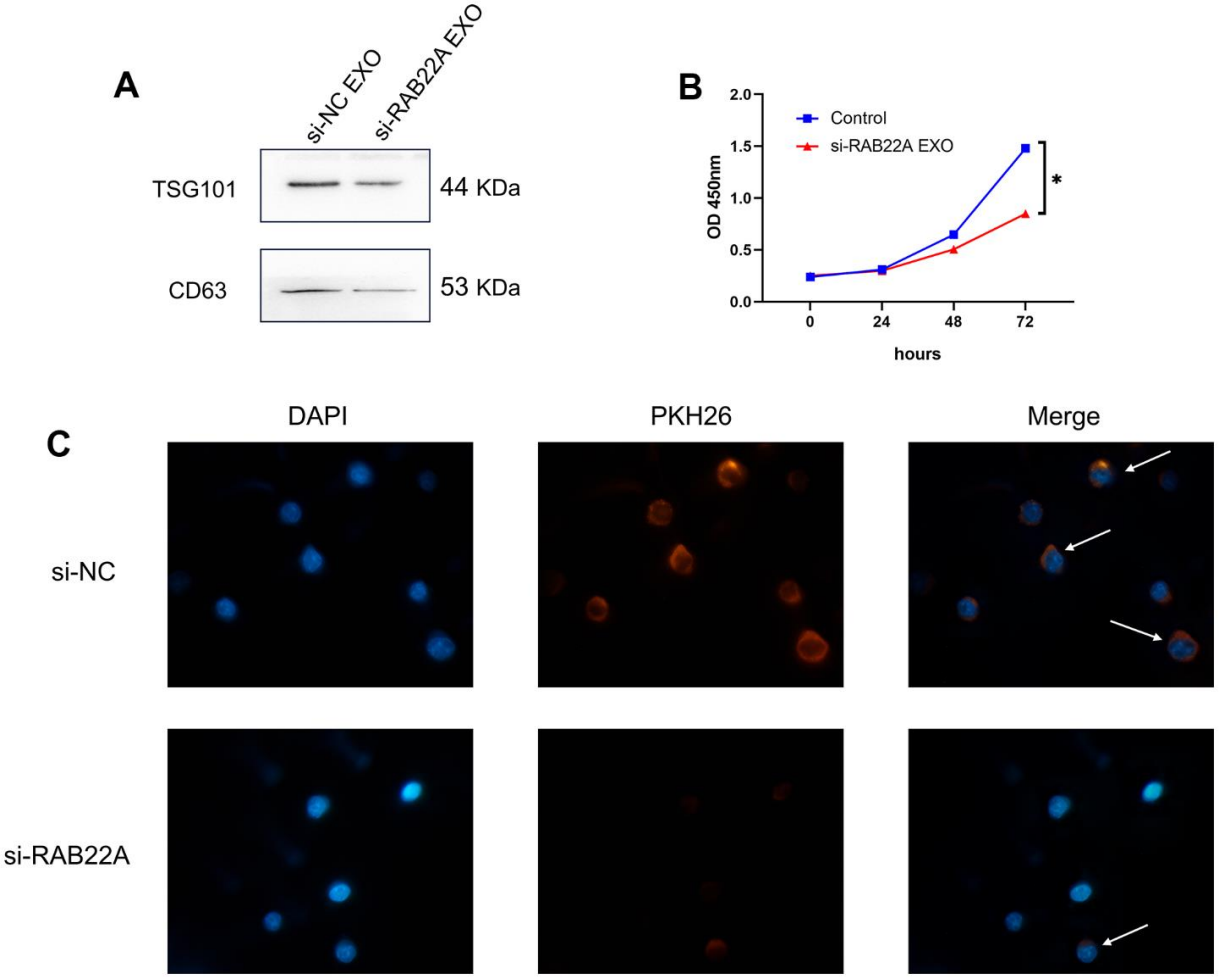
**Effects of regulating RAB22A on exosomes secretion and cell proliferation.** (**A**) Verification of exosomes by western blotting. (**B**) The exosomes of MSCs in control group and down-regulated RAB22A group were co-cultured with MM cell line, and the OD values of MM cells at different time points were detected by CCK-8 method. (**C**) The exosomes of control group and down-regulated RAB22A group were collected and stained with PKH26. The stained exosomes were co-cultured with MM cells, and the endocytosis of exosomes was observed under fluorescence microscope.

We collected the clinical information of 71 normal controls and 30 newly diagnosed MM patients. After 1: 1 propensity score matching (PSM) was performed to eliminate the bias caused by sex and age, 25 normal controls and 25 newly diagnosed MM patients were included for follow-up analysis (Supplementary Table 4). We used western blot and qRT-PCR to verify the difference in the expression level of RAB22A. The results showed that the expression level of RAB22A in MM was significantly higher than that in normal people (Figure 10A, 10B, 10E), and the expression level in MM-BMSC was significantly higher than that in Normal-BMSC (N-BMSC) (Figure 10C, 10D, 10F). Then, we down-regulated RAB22A expression in U266, RPMI 8226 cell lines and MM-MSCs by lentivirus transfection. After down-regulating RAB22A in MM cell line, the cell proliferation was significantly decreased (Figure 10G). We extracted the exosomes of MSCs from the control group and the si-RAB22A group, identified them by exosome markers (Figure 11A), and co-cultured the exosomes from MSCs with MM cell lines. Immunofluorescence staining results showed that the secretion of exosomes decreased after inhibiting the expression of RAB22A in MSCs (Figure 11C). The results of CCK-8 assay showed that compared with the exosomes of MSCs that down-regulated RAB22A, the exosomes of MSCs in control group significantly promoted the proliferation of MM cells (Figure 11B).

## DISCUSSION

Multiple myeloma is a hematological tumor caused by malignant clonal proliferation of bone marrow plasma cells, which is largely caused by the imbalance of oncogenes and abnormal immune system [26]. MM developed from precancerous disease MGUS, and gradually developed into SMM and MM at the rate of 1% per year [27]. RAB22A belongs to the RAS oncogene family, and its abnormal overexpression is involved in the occurrence and progress of many cancers [15, 17]. The significance and mechanism of RAB22A expression in MM are not clear. This study found that RAB22A was significantly overexpressed in SMM and MM, which may lead to the disease progression of MM by regulating exosome secretion, EMT and immune function.

Although the initiation of MM is mainly related to the abnormal activation of key genes and pathways, the support of bone marrow microenvironment is also an important factor leading to the malignant progress of plasma cells. The seed and soil theory in cancer also proves the importance of the interaction between tumor cells and microenvironment for tumor progression and drug resistance. Abnormal bone marrow microenvironment provides protection for tumor cells, which makes drug-resistant tumor cells lurk in it, forming minimal residual disease, and then becomes the source of tumor recurrence and drug resistance. MSCs are one of the important contributing factors in bone marrow microenvironment, and they are a kind of progenitor cells with self-renewal ability and multi-lineage differentiation potential, which can be differentiated into adipocytes, endothelial cells, osteoblasts and fibroblasts [28]. Although MM cells interact with many bone marrow cells, it has been reported that BM-MSCs are the extremely key components to promote the growth, proliferation and drug resistance of MM cells [29]. Cell-to-cell communication between MSCs and MM is necessary for the progress of MM. The invasion of MM cells into bone matrix changed the expression profile of miRNAs in MSCs, which further affected the osteogenic differentiation direction of MSCs, so that osteoclasts were activated and osteoblasts were inhibited, resulting in osteolytic lesions [30–32]. Exosomes are a subtype of membrane-bound extracellular vesicles released by various cells, with a diameter of about 30-150nm. They are rich in mRNAs, miRNAs, proteins and lipids, and can be used for substance exchange and signal transmission between cells [33]. The number of exosomes secreted by tumor cells is much higher than that of normal cells, and the amount of exosomes is related to the malignant degree of tumors. Notably, the gene data carried by RNA directly reflects the protein expression and biological activity of the target cell [34]. The content of miRNAs in exosomes secreted by MM-MSCs is different from that secreted by normal MSC, which leads to the proliferation of MM cells and promotes the disease progression [35]. Another study found that compared with the exosomes of normal MSCs, the expression of miR-483-5p in the exosomes of MM-MSCs was significantly increased, which promoted the proliferation of MM cells and reduced apoptosis by acting on the downstream target TIMP2 [36]. Previous studies have proved that exosome-mediated intercellular communication promotes the malignant progress of MM, but the factors that regulate the exosome secretion of MSCs are not clear. In this study, we co-cultured the exosomes of MM-MSCs from the control group and si-RAB22A group with MM cells. The results of CCK-8 assay showed that the exosomes of MM-MSCs from si-RAB22A group reduced the proliferation of MM. Therefore, it is very important to further explore the genes that affect the secretion of exosomes for the study of drug resistance mechanism and the development of drug targets.

RAB22A is highly expressed in colorectal cancer, hepatocellular carcinoma and breast cancer, and participates in extracellular vesicle secretion, EMT and immune regulation. RAB22A transports the contained substance to the right place by participating in the formation of early endosomes and controlling the formation, movement and fusion of vesicles [37]. In one study [16], RAB22A promoted the formation of extracellular vesicles containing STING, so that STING was transported to the tumor microenvironment. According to literature review, there is no research on the effect of RAB22A on the exosome secretion of MSCs in MM at present, which urges us to pay attention to the effect of RAB22A on the exosome secretion and intercellular communication. In this study, we analyzed the difference of gene expression profile of MSCs between newly diagnosed and relapsed MM patients, and found that the expression level of RAB22A in relapsed patients was significantly higher than that in newly diagnosed patients, and it was related to exosome-related genes and exosome scores. This may suggest that the overexpression of RAB22A promotes the secretion of exosomes in MSCs, which led to the recurrence of MM. Therefore, we further regulated the expression level of RAB22A in MSCs. The results showed that the secretion of exosomes decreased after inhibiting the expression of RAB22A of MM-MSCs, and compared with the exosomes derived from MM-MSCs in the control group, the proliferation of MM cells was significantly inhibited.

EMT is one of the characteristics of solid tumor metastasis, and EMT phenotype also exists in many hematological tumors. A study found that S100A4 was associated with the low survival rate of pancreatic cancer, and TGFβ1 promoted EMT and increased invasiveness by up-regulating S100A4 expression level [38]. In addition, CXCR4 was highly expressed in MM patients with extramedullary invasion, which promoted cell spread and metastasis by inducing EMT phenotype [39]. Another study found that the expression of NSD2 was significantly higher in MM cells with t (4,14) chromosome translocation, and the expression of EMT-related genes was increased by up-regulating transcription factor TWIST1, thus promoting the migration and spread of MM cells [40]. Previous studies have found that RAB22A participates in EMT process in melanoma and colorectal cancer, leading to malignant development of tumor [17, 18]. We analyzed the EMT-related genes that were significantly differentially expressed between the high and low RAB22A expression groups, and calculated the EMT scores of the two groups by “PCA” algorithm. Compared with the low RAB22A expression group, the expression levels of EMT-related genes MYBL2, ITGB1, CDK5, CBR1, EPCAM, S100A4, FOXM1, TIMP1, BHLHE40 and AHR in the high RAB22A expression group were significantly higher, and they had higher EMT scores.

The tumor microenvironment is mainly composed of tumor cells, immune cells, stromal cells and non-cellular components. The components in the tumor microenvironment interact with tumor cells, affecting the occurrence, curative effect and prognosis of tumors. MDSCs are a group of heterogeneous immature myeloid cells with strong immunosuppressive function. The increase of MDSCs leads to poor efficacy of immunotherapy, poor prognosis and disease progression. The expression of MDSCs is increased in hematological tumors, which promotes immune tolerance by inhibiting T cell proliferation. In AML [41], MUC1 induced an increase in the expression of c-myc in extracellular vesicles, which acted on the cyclin of MDSCs, thus promoting the proliferation of MDSCs. In MM, MDSCs promoted the proliferation of MM cells, and played an immunosuppressive role by inhibiting T cell function and Treg cells amplification [42]. Treg cells are a subset of T cells with significant immunosuppression, characterized by the expression of Foxp3, CD25 and CD4, which are related to the disease progression and poor prognosis of multiple myeloma [43, 44], lung cancer [45], hepatocellular carcinoma [46] and breast cancer [47, 48]. Treg cells inhibits the immune response of the body, leading to immune escape of tumor cells [49, 50]. Therefore, Treg cells are considered as the key target of cancer immunotherapy [51]. In our study, the expression level of RAB22A was positively correlated with Treg cells infiltration level, and the infiltration degree of MDSCs in high RAB22A expression group was higher than that in low RAB22A expression group. These results suggested that RAB22A played an important role in regulating immune cell infiltration of MM.

Although this study explored the expression level and biological function of RAB22A in MM through bioinformatics analysis and preliminary verification of clinical samples, there are still some shortcomings. First of all, the transcriptome data of this study came from public databases, which needs to be further verified in clinical samples in the future. Secondly, this study lacked follow-up data and specific treatment information, such as treatment response, survival time, radiotherapy and chemotherapy information, which were very important for the construction of prognosis model. We will further follow up and analyze the prognosis in the clinical samples of our center. Finally, this study did not further analyze the complete transcriptome of exosomes secreted by MSCs. By analyzing the difference of substances transported by exosomes, we will further explore the downstream effector molecules of RAB22A, which is expected to develop new targets in MM. This is what we will continue to explore in the future.

In a word, we confirmed that RAB22A was highly expressed in MM and SMM. RAB22A is related to EMT, exosome secretion, immune cell infiltration and m6A modification. We verified *in vitro* that RAB22A regulated the proliferation of MM by affecting the secretion of exosomes. RAB22A may be a potential therapeutic target to improve the prognosis of MM.

## Supplementary Material

Supplementary Figure 1

Supplementary Tables
