# Supplementary material for: RAB22A as a predictor of exosome secretion in the progression and relapse of multiple myeloma
Source: Aging (Albany NY). 2024 Mar 1;16(5):4169–90. doi: 10.18632/aging.205565 (PMC10968671; doi:10.18632/aging.205565)
Supplement: Supplementary Figure 1 [file aging-16-205565-s001.pdf]

## SUPPLEMENTARY FIGURE

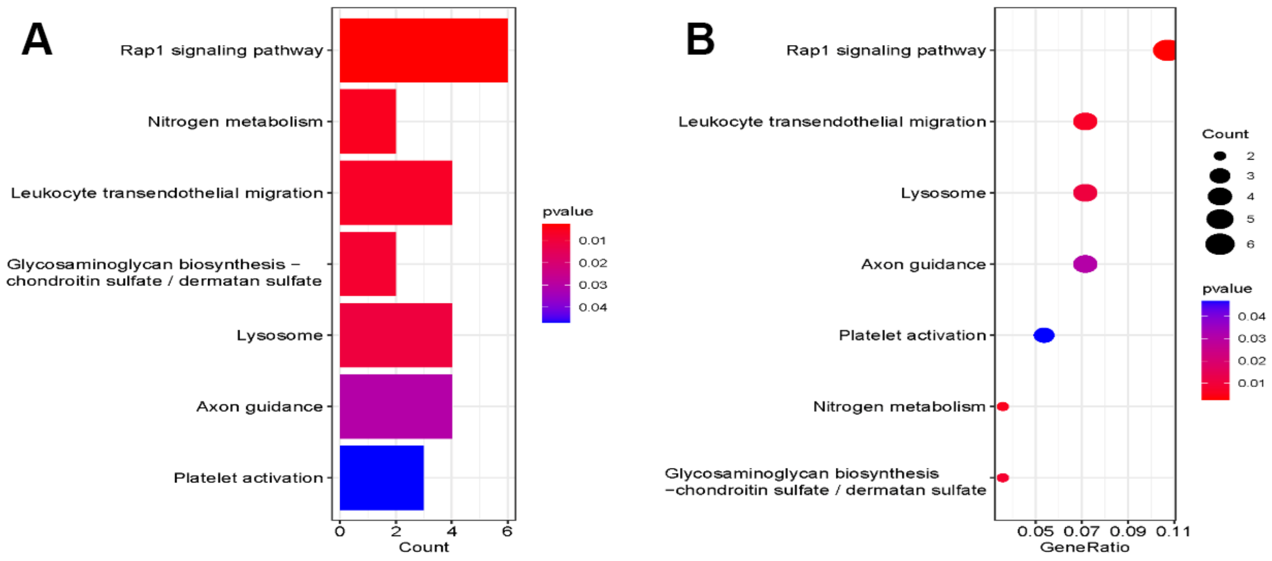


**Supplementary Figure 1.** (**A**, **B**) KEGG enrichment analysis was performed on differentially expressed genes.
