# Supplementary material for: RAB22A as a predictor of exosome secretion in the progression and relapse of multiple myeloma
Source: Aging (Albany NY). 2024 Mar 1;16(5):4169–90. doi: 10.18632/aging.205565 (PMC10968671; doi:10.18632/aging.205565)
Supplement: Supplementary Tables [file aging-16-205565-s002.pdf]

SUPPLEMENTARY TABLES

Supplementary Table 1. Exosome-related genes.

Gene
YWHAG
YWHAQ
CLTC
NCKAP1
CFL1
ACTB
CCT4
RDX
GNA13
CTNNB1
TSG101
CD9
CD81
CD63
FLOT1
ITGB1
ITGA1
HSP70
PDCD6IP
RAB27A
RAB27B
PIKFYVE
HRS
SYT7
CTTN
STAT3
PKM2
UNC13D
EGFR
RAS
EIF3C
STK11
YWHAG

Supplementary Table 2. Immune checkpoint genes.

Gene
BTNL3
BTNL9
C10orf54
CD209
CD226
CD27
CD274
CD276
CD47
CD80
CTLA4
HAVCR2
HLA-B
HLA-DMA
HLA-DRA
LAG3
PDCD1
PDCD1LG2
PVR
SIRPA
TDO2
TIGIT
TNFRSF14
TNFRSF4
TNFSF14

Supplementary Table 3. M6A-related genes.

Gene	Type
METTL3	writers
METTL14	writers
VIRMA	writers
RBM15	writers
RBM15B	writers
CBLL1	writers
HNRNPC	readers
FMR1	readers
HNRNPA2B1	readers
IGFBP1	readers
IGFBP2	readers
IGFBP3	readers
FTO	erasers
ALKBH5	erasers

Supplementary Table 4. PSM analysis.

Match type	Count
Exact Matches	4
Fuzzy Matches	21
Unmatched Including Missing Keys	46
Unmatched with Valid Keys	46
